# Spindle assembly checkpoint genes reveal distinct as well as overlapping expression that implicates MDF-2/Mad2 in postembryonic seam cell proliferation in *Caenorhabditis elegans*

**DOI:** 10.1186/1471-2121-11-71

**Published:** 2010-09-21

**Authors:** Maja Tarailo-Graovac, Jun Wang, Jeffrey SC Chu, Domena Tu, David L Baillie, Nansheng Chen

**Affiliations:** 1 Department of Molecular Biology and Biochemistry, Simon Fraser University, Burnaby, British Columbia, V5A 1S6, Canada

## Abstract

**Background:**

The spindle assembly checkpoint (SAC) delays anaphase onset by inhibiting the activity of the anaphase promoting complex/cyclosome (APC/C) until all of the kinetochores have properly attached to the spindle. The importance of SAC genes for genome stability is well established; however, the roles these genes play, during postembryonic development of a multicellular organism, remain largely unexplored.

**Results:**

We have used GFP fusions of 5' upstream intergenic regulatory sequences to assay spatiotemporal expression patterns of eight conserved genes implicated in the spindle assembly checkpoint function in *Caenorhabditis elegans*. We have shown that regulatory sequences for all of the SAC genes drive ubiquitous GFP expression during early embryonic development. However, postembryonic spatial analysis revealed distinct, tissue-specific expression of SAC genes with striking co-expression in seam cells, as well as in the gut. Additionally, we show that the absence of MDF-2/Mad2 (one of the checkpoint genes) leads to aberrant number and alignment of seam cell nuclei, defects mainly attributed to abnormal postembryonic cell proliferation. Furthermore, we show that these defects are completely rescued by *fzy-1(h1983)/CDC20*, suggesting that regulation of the APC/C^CDC20 ^by the SAC component MDF-2 is important for proper postembryonic cell proliferation.

**Conclusion:**

Our results indicate that SAC genes display different tissue-specific expression patterns during postembryonic development in *C. elegans *with significant co-expression in hypodermal seam cells and gut cells, suggesting that these genes have distinct as well as overlapping roles in postembryonic development that may or may not be related to their established roles in mitosis. Furthermore, we provide evidence, by monitoring seam cell lineage, that one of the checkpoint genes is required for proper postembryonic cell proliferation. Importantly, our research provides the first evidence that postembryonic cell division is more sensitive to SAC loss, in particular MDF-2 loss, than embryonic cell division.

## Background

The spindle assembly checkpoint (SAC) acts as a surveillance mechanism by delaying the metaphase-to-anaphase transition until all the chromosomes have properly aligned and attached to the mitotic spindle; thus, preventing chromosome instability (CIN). In the presence of even a single improperly attached kinetochore, SAC is activated to inhibit a large multisubunit E3 ubiquitin ligase complex, the anaphase promoting complex/cyclosome (APC/C), and prevents anaphase onset [1]. APC/C activity requires the association of Cdc20 in early mitosis, while Cdh1 (encoded by *Fzr1 *in mammals) is required to activate APC/C in late mitosis and during G1 [2,3]. The primary target of SAC is the Cdc20 activator that, when inhibited, cannot activate APC/C to degrade securin [1]. Degradation of securin is required for activation of separase and cleavage of cohesion between sister chromatids which in turn triggers anaphase onset in mitotic cells [1].

The first identified components of SAC were isolated in two independent genetic screens in *Saccharomyces cerevisiae *and include *MAD1*, *MAD2*, *MAD3*, *BUB1*, and *BUB3 *[4,5]. These proteins are widely conserved, both structurally and functionally, throughout the eukaryotic kingdoms [1]. However, additional proteins essential for the checkpoint activity have continued to be discovered in higher eukaryotes. These include Rod (ROugh-Deal), Zw10 (Zeste-White 10) and CENP-F proteins, among others [6-8]. These components lack clear yeast orthologs, suggesting that, in higher eukaryotes, checkpoint signaling is more elaborate.

The SAC components and the checkpoint signalling pathway are highly conserved in *C. elegans*. The *C. elegans *homologues of the SAC components, originally discovered in yeast, have been identified and named *mdf-1*, *mdf-2*, *san-1*, *bub-1 *and *bub-3*, respectively [9-13]. Recent availability of knockout alleles of these checkpoint components, in addition to RNA interference (RNAi) experiments, allowed assessment of the phenotypic consequences in the absence of the SAC gene products [9,12,14]. All of these genes are important for genome stability and viability in the presence of spindle damage [9,11,12,15]. However, while *mdf-2*, *san-1 *(known as *MAD3 *in other systems) and *bub-3 *become essential only in the presence of chemical or mutational disruptions of the mitotic spindle [9,11,12,15], *bub-1 *and *mdf-1 *are essential for embryonic viability, long-term survival and fertility under normal laboratory conditions in *C. elegans *[9,16]. In fact, analysis of an *mdf-1 *deletion mutant, *mdf-1(gk2)*, gave the first demonstration of what affect a defective checkpoint has on animal development [9]. In the absence of MDF-1, severe developmental defects are observed, including embryonic lethality, larval arrests, abnormal vulva development, and sterility, which lead to lethality of the homozygous strain after three generations [9]. Similar developmental defects have also been observed in the absence of MDF-2 [9,12]; however, unlike Δ*mdf-1 *animals, Δ*mdf-2 *homozygotes can be propagated indefinitely [12]. The fact that absence of different SAC components leads to different developmental consequences in *C. elegans*, as well as other organisms [17,18], suggests differential requirement of these genes in development and fertility that may or may not be distinct from their function in SAC.

To investigate roles SAC genes have during postembryonic development of a multicellular organism, we studied spatiotemporal expression patterns of the checkpoint genes. As expected, SAC promoters drive mainly ubiquitous GFP expression during early embryonic development. However, all SAC promoters drive tissue-specific expression in later developmental stages. Further analysis revealed that the MDF-*2 *checkpoint component is required for proper postembryonic proliferation of seam cells by regulating APC/C^CDC20^. In fact, seam cell proliferation was abrogated at a higher frequency during the proliferative L2 stage than in the embryo, suggesting that postembryonic cell divisions may be more sensitive to loss of the checkpoint than the embryonic cell divisions. Furthermore, we showed that while the hypomorphic mutant *fzy-1*(*CDC20*) fully restored proper seam cell proliferation; *fzr-1*/*CDH1 *mutant had no effect on seam cell development in a Δ*mdf-2 *background.

## Results

### Generation of pSAC::GFP *C. elegans *strains and characterization of SAC expression patterns

In order to explore the temporal and spatial expression of SAC genes, we generated transcriptional reporter transgenic *C. elegans *strains for the five widely conserved checkpoint core components (*mdf-1/MAD1*, *mdf-2/MAD2*, *san-1/MAD3*, *bub-1/BUB1 *and *bub-3/BUB3*) and four SAC components only conserved in higher eukaryotes (*hcp-1/CENP-F*, *hcp-2/CENP-F, czw-1/ZW10 *and *rod-1/ROD*) (Table 1). All of the selected genes, except for *mdf-1*, are not in operons, and thus sequences immediately upstream were used for their promoter analysis. *mdf-1*, on the other hand, is part of an operon and was probed using three different promoter constructs (Table 1, Additional file 1, Figure S1). The promoter::GFP fusions were generated using a "PCR stitching" technique [19], rather than by cloning methods, to avoid potential interference from cloning vector backbones on transgene expressions, as reported recently by Etchberger and Hobert, 2008 [20]. The putative "promoter" amplicons were "PCR-stitched" to the PCR products containing a gfp encoding sequence (S65C variant) that includes artificial introns and the *unc-54 *3'UTR from the pPD95.75 vector (developed by Dr. Andrew Fire, Carnegie Institution). The 5' regions examined in this study as putatively containing regulators of the SAC genes extended from the predicted ATG initiator site for a targeted gene to its adjacent upstream gene. The lengths of the upstream regions defined by these criteria range in size from 282 bp to 3,000 bp (Table 1) and are in accordance with previously described minimum and maximum promoter lengths used in large scale projects [21-25]. In total, 12 transcriptional fusions with gfp were constructed corresponding to the nine checkpoint genes of interest (Table 1). For each construct, we generated at least three independent lines that were compared for expression pattern consistencies. Due to the mosaicism issues associated with extrachromosomal concatameric arrays, we analyzed at least 30 replicates and recorded GFP-expressing cells and tissues that showed expression in at least 50% of the animals at any given developmental stage, as described previously [23].

**Table 1:** Summary of postembryonic spatial GFP expression observed for SAC gene transcriptional reporters

Gene	5' Regulatory Region Size (bp)	Nervous System	Intestine	Pharynx	Gonad	Hypodermis	Seam cells	Vulva	Coelomocytes
C50F4.11 (*mdf-1*)	295 ^1^		X	X					
C50F4.11 (*mdf-1*)	543 ^2^		X						
C50F4.11 (*mdf-1*)	1332 ^3^		X						
Y69A2AR.30 (*mdf-2*)	301		X	X		X	X	X	
ZC328.4 (*san-1*)	597		X			X	X		
R06C7.8 (*bub-1*)	1130	h	X	X		X	X	X	
Y54G9A.6 (*bub-3*)	282		X	X		X	X	X	
ZK1055.1 (*hcp-1*)	591	d,v,h,t,b	X	X	s,u	X	X	X	X
T06E4.1 (*hcp-2*)	427	t	X	X	dtc	X	X		
F20D12.4 (*czw-1*)	2101								
F20D12.4 (*czw-1*)	3000								
F55G1.4 (*rod-1*)	1163		X						

5' Regulatory Region column lists the corresponding sizes of the putative promoters used to drive GFP expression.

X = expression observed; h = head neurons; d = dorsal nerve cord; v = ventral nerve cord; t = tail neurons; b = mid-body neurons; s = spermatheca; u = uterus; dtc = distal tip cells.

^1 ^Upstream region, from the ATG initiator site in the first gene, *his-35*, of the *mdf-1 *containing operon to an adjacent upstream gene *his-41*.

^2 ^Upstream region from the ATG initiator site in *mdf-1 *to an adjacent upstream gene *his-35*.

^3 ^Upstream region from the ATG initiator site in *mdf-1 *to the operon upstream gene *his-41*.

Our analysis of SAC gene regulatory activities revealed that all of the SAC constructs, except for p*czw-1*::GFP, confer GFP expression (Table 1). The 2,101 bp sequence upstream of *czw-1 *did not drive any detectable GFP expression at any developmental stage in any of the four independent transgenic lines analyzed. We also examined another construct that contained 3 kb upstream sequence of *czw-1 *and still did not observe any expression (Table 1). Importantly, our analysis of the other eight SAC genes revealed expression that was consistent between the independent lines for every given construct (Table 1). We have detected GFP at all developmental stages, except for very young embryos (younger than 12 cell-stage embryos), and have identified expressed GFP in all the major tissues, except for germline, likely due to germline silencing of concatameric arrays [26].

### Promoters of spindle assembly checkpoint genes drive similar early embryonic expression

**Figure 1:**
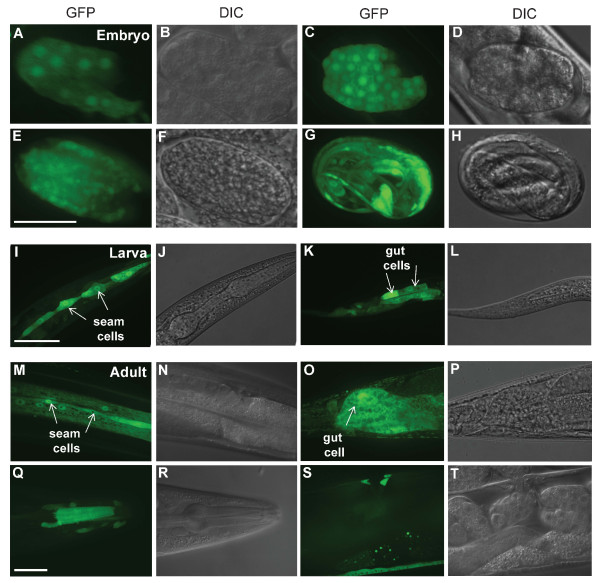
**Expression patterns driven by the *mdf-2 *promoter**. p*mdf-2*::GFP is expressed at all developmental stages of *C. elegans*. Representative images of the pre-comma (A-D), mid (E and F) and late (G and H) embryonic stages with the majority of cells expressing GFP. (I-L) Representative images of the GFP signal in seam cells at L3 (I and J) and gut cells at L2 (K and L). (M-T) Representative images of the adult tissues that contain GFP signal including seam (M and N), gut (O and P), pharyngeal (Q and R) and vulval cells (S and T). The scale bar represents 25μm.

**Figure 2:**
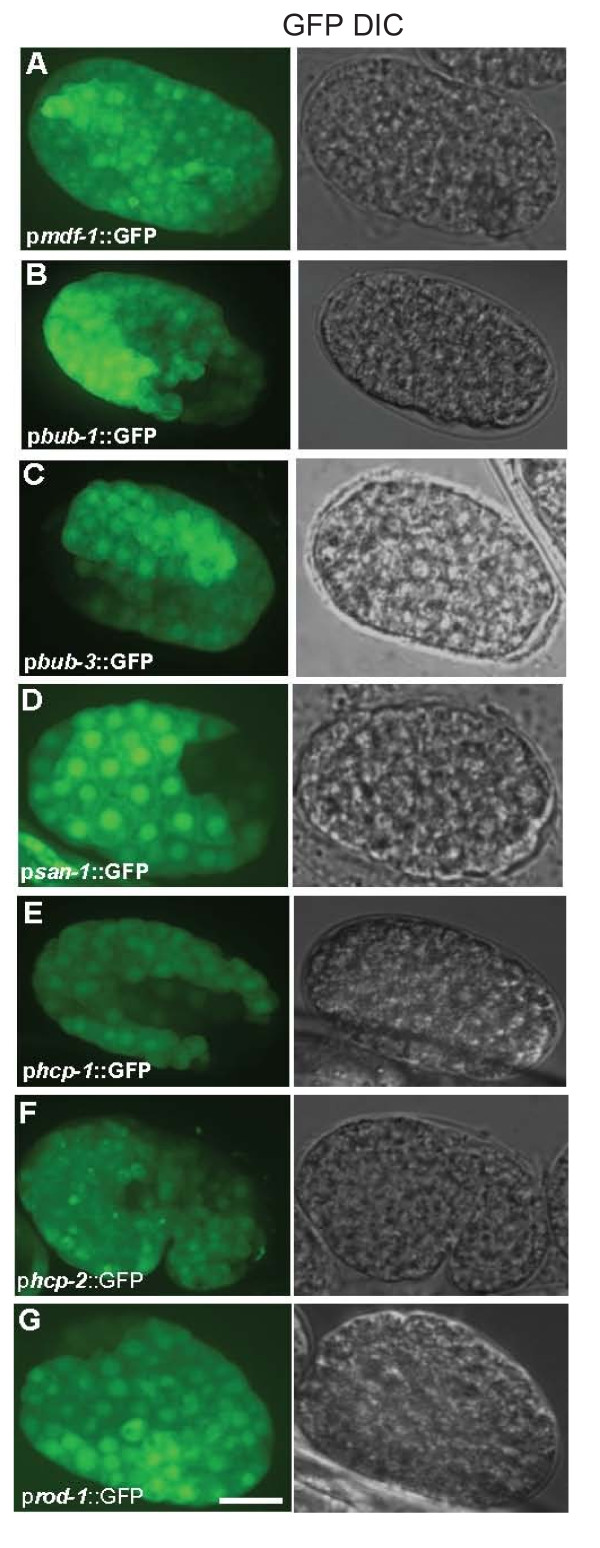
**Embryonic expression of seven spindle assembly checkpoint genes**. (A-G) Representative images of early embryonic (pre-comma stage) SAC promoter activities; GFP and DIC images are shown. (A) For *mdf-1*, the 1332 bp 5' regulatory region that contains the internal promoter is depicted. (F) For the *hcp-2 *promoter, bean stage embryo is shown. Notice that in some images, like (E), clear mosaicism is observed. Some nuclei have weak GFP signal, while some have no detectable signal. This is likely due to loss of the array. The scale bar represents 10μm.

GFP expression driven by the eight SAC gene upstream regions containing regulatory sequences (promoters) was commonly observed early in development, well before the comma stage of embryogenesis (Figures 1A-D and 2). In fact, we were able to detect GFP expression before embryos progressed to gastrulation (Figure 1A). Because we observed mosaicism due to mitotic loss of the concatamer arrays (Figures 1 and 2), we analyzed many embryos per construct. Our results show that SAC gene promoters drive GFP expression in the majority of the early embryonic cells (Figures 1A-D and 2). The only construct that did not drive ubiquitous GFP expression in early embryos is the putative promoter of *mdf-1*, which is in an operon, that extends upstream from the ATG initiator site in the first gene, *his-35*, of the operon to the adjacent upstream gene (*his-41*) (Additional file 1, Figure S1A). On the other hand, both transcriptional fusions that included an internal *mdf-1 *promoter revealed the same ubiquitous activities in early embryos (Additional file 1, Figure S1B, C). Considering the established role of the *mdf-1 *checkpoint gene in surveillance of the metaphase-to-anaphase transition, as well as the observed antibody localization in dividing cells in early embryos [27], we conclude that the *mdf-1 *containing operon belongs to the "hybrid operons" class [28], in which the internal promoter of *mdf-1 *is necessary to drive proper expression of this gene in embryonic cells.

The cell cycles of early embryonic cells in *C. elegans *are rapid, consist entirely of S phase and mitosis, and lack gap phases [29]. This rapid embryonic cell proliferation creates more than half of *C. elegans*' somatic cells, with the majority of cell divisions being completed in the first half of embryogenesis [30]. Thus, co-expression of SAC genes in the rapidly dividing early embryonic cells (Figures 1A-D and 2) is consistent with the well established role of these genes in cell division. In addition to the activities of SAC gene promoters in the early embryos, we also observed GFP expression in later embryos for all of the spindle-checkpoint promoters that we analyzed. The expression patterns in late embryos show GFP expression in the majority of the cells, although the majority of the promoter constructs tend to confer more localized GFP expression, as exemplified for *mdf-2 *(Figure 1G). Together, the expected promoter activities of SAC genes during embryogenesis, show that the promoters used for our analysis are appropriate.

### SAC promoters drive tissue-specific gene expression later in development

Rapid cell proliferation occurs in all four larval stages especially in the second larval stage (L2) of development in *C. elegans *when many somatic cells are generated [30]. As expected, GFP expression conferred by SAC gene promoters was detected at all four larval stages (Table 1). Unlike embryonic expression, spatiotemporal analysis revealed that postembryonic expression of SAC genes is generally restricted to specific cells and tissues types (Table 1). For example, *mdf-2 *promoter drives GFP expression in seams cells (Figure 1I), gut cells (Figure 1K), and some additional tissue types (Table 1) at all larval stages. In contrast, *mdf-1*^internal ^and *rod-1 *promoters drive GFP expression specifically in gut cells after embryogenesis (Table 1). Unlike *mdf-2*, *mdf-1* and *rod-1* promoters, *hcp-1 *promoter was found to be active in the majority, but not all, tissues analyzed, including dorsal/ventral nerve cord, head/tail/body neurons and many other tissue types (Table 1). Thus, postembryonic spatial analysis revealed distinct, yet overlapping, tissue-specific expression of SAC genes during larval development.

**Figure 3:**
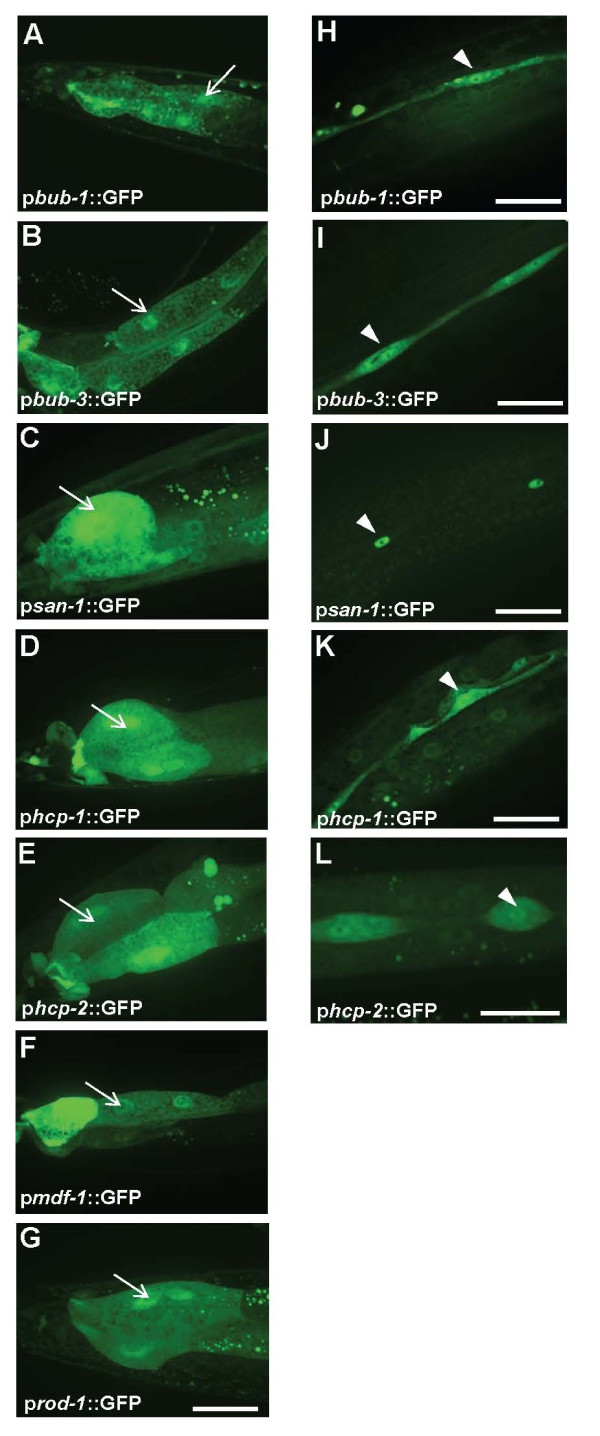
**Co-expression of the SAC genes in gut and seam cells of the adult animals**. (A-G) All of the SAC promoters drive GFP expression in gut cells, depicted by *arrows*. (H-L) The majority of the SAC gene promoters drive GFP expression in seam cells, depicted by *arrowheads*. The scale bar represents 25μm.

Unexpectedly, we also observed tissue-specific expression of SAC genes at late larval (late L4) and adult stage (Figures 1M-T and 3). Since there are no cell divisions during late L4 and at adulthood except for the divisions in somatic gonads that lead to oocyte development [30], our observations suggest that SAC genes are expressed in non-proliferating cells in *C. elegans*. Similar to larval expression profiles, tissue-specific expression is observed in adult animals as well. For example, as in larvae, *mdf-2 *promoter drives GFP expression in seam cells and hypodermis (Figure 1M), gut cells (Figure 1O), pharynx (Figure 1Q), and vulva (Figure 1S). The expression patterns detected in adult tissues further support the striking co-expression of the checkpoint genes in hypodermal seam cells (Figures 3H-L) and intestine (Figures 3A-G) that we observed in larval stages.

### Absence of MDF-2 results in aberrant number and alignment of seam cell nuclei

We were interested in testing whether absent or nonfunctional SAC would cause aberrant postembryonic seam cell development. For this analysis, we chose *mdf-2*. MDF-2 shares 40% sequence identity with budding yeast Mad2 and rescues benomyl sensitivity of the *mad2 *knockout strain in yeast, suggesting functional checkpoint conservation [9]. Like *Δmdf-1*, absence of MDF-2 leads to severe defects in larval and germ cell development, suggesting essential roles in postembryonic development [9,12]. Unlike *Δmdf-1*, knockout strain of *mdf-2 *is viable [12].

**Figure 4:**
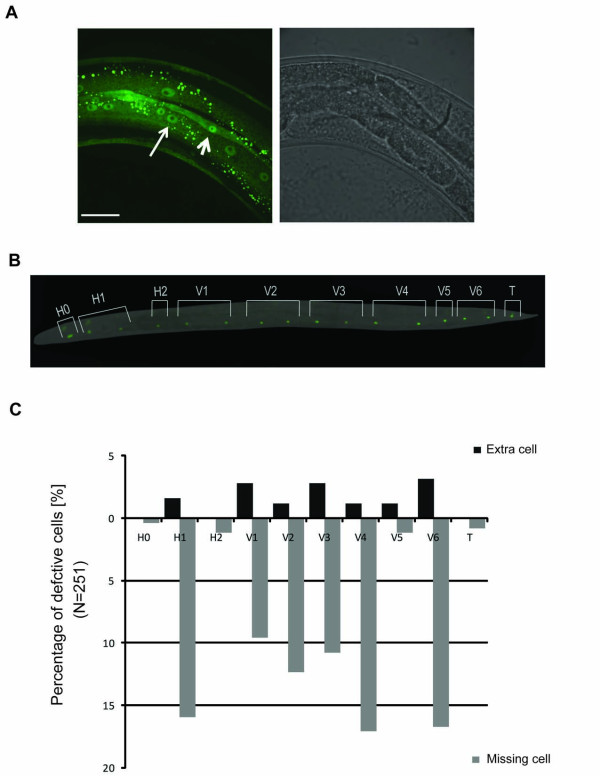
***mdf-2/*MAD2 (a spindle-checkpoint gene) is expressed in hypodermal seam cells and is important for their proper development**. (A) Expression driven by *mdf-2 *promoter in hypodermis (*long arrow*) and seam cells (*short arrow*) using the multi-copy stable line JNC116. The scale bar represents 25μm. (B) An adult wild-type worm containing 16 SCM::GFP nuclei, originating from H0-H2, V1-V6, and from the T seam cell. (C) Quantitative analysis of 251 animals with the seam cell defect. Black bars represent percentage of extra cells observed in individual seam cells, while the grey bars represent percent of the individual seam cells that are missing in the defective animals.

Our spatiotemporal analysis using extra-chromosomal concatameric arrays revealed that the promoter of *mdf-2 *drives expression of the GFP reporter in hypodermis and seam cells (Figures 1I and 1M), and some other cell types. We also constructed two chromosomal integrant p*mdf-2::GFP *strains, a multi-copy stable line (putatively integrated into the genome), and a stable line generated using the recently developed Mos1-mediated Single-Copy Insertion (MosSCI) method [31]. Using the multi-copy stable line, we observed similar expression patterns in hypodermis and seam cells (Figure 4A), and other cell types. MosSCI method, on the other hand, allows integration of transgenes as single copies at a few specific loci in *C. elegans*' genome. Although the p*mdf-2::GFP *stable line generated using MosSCI had > 10 × lower intensity of the GFP expression than the multi-copy stable line (data not shown), it further confirmed the expression patterns that we observed using a p*mdf-2::GFP *extrachromosomal transgene in postembryonic hypodermis and seam cells (data not shown).

**Figure 5:**
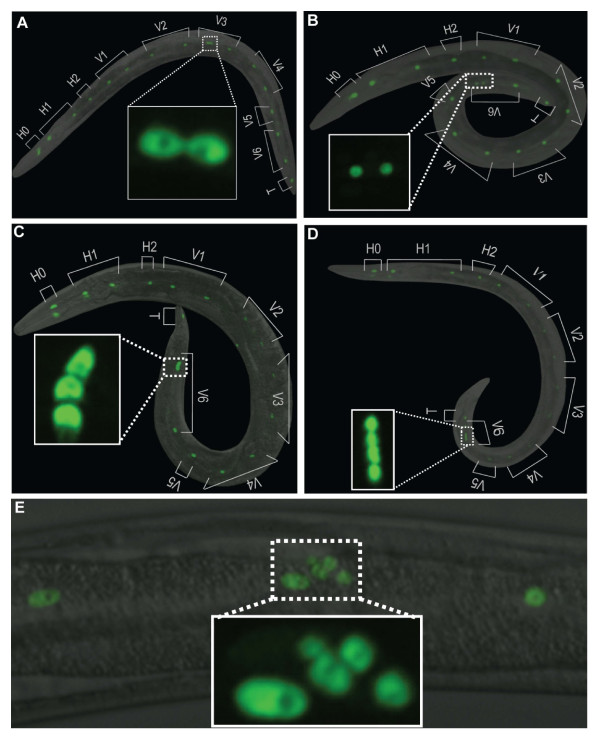
**Representative images of extra SCM::GFP positive nuclei observed in the *mdf-2(tm2190*) animals**. (A) An animal has 17 positive SCM::GFP nuclei. It appears to have one extra V3 seam cell with two fused together. (B) An animal has 17 positive SCM::GFP nuclei. It appears to have one extra V6 seam cell. (C) An animal has 18 positive SCM::GFP nuclei. It appears to have two extra V6 seam cells. (D) An animal has 19 positive SCM::GFP nuclei. It appears to have three extra V3 seam cells. (E) An example of five SCM::GFP positive nuclei in the region where only a single seam cell should reside. The animals depicted are in either L4 or young adults.

**Figure 6:**
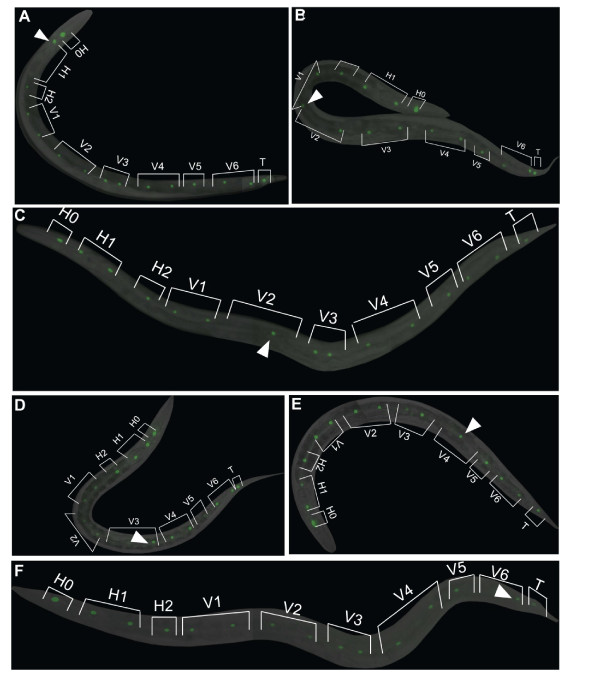
**Representative images of *mdf-2(tm2190*) homozygotes with only 15 SCM::GFP positive nuclei**. (A) H1 seam cell appears to be missing. (B) An example of ambiguous case where either V1 or V2 seam nucleus is missing. (C) V2 seam cell appears to be missing. (D) V3 seam cell appears to be missing. (E) V4 seam cell appears to be missing. (F) V6 seam cell appears to be missing. Arrowheads depict a single seam nucleus in the lineages where two seam nuclei are expected to be observed.

To determine the consequence of absence of MDF-2 on normal seam cell development, we examined and quantified the number of seam cell nuclei in transgenic strains expressing SCM::GFP [32] (*s*eam *c*ell *m*arker fused to GFP) in the *mdf-2(tm2190*) knockout, *Δmdf-2*, background using fluorescence microscopy (Figures 4B, 5 and 6). The *tm2910 *deletion removes 864 nucleotides between intron 3 and exon 6 and is likely to be a null mutation. The SCM::GFP marker allows visualization of the number of seam cell nuclei and their morphology during development. Our analysis of young adult animals homozygous for *Δmdf-2 *revealed both qualitative and quantitative difference compared to wild-type animals (Figures 4-6; Table 2). While wild-type adult hermaphrodites usually contain 16 evenly spaced and aligned SCM::GFP nuclei on each side of the animals [32] (Figure 4B), *Δmdf-2 *adult hermaphrodites frequently have non-aligned seam cell nuclei clustered in one part of the body (Figures 5 and 6). Such clustering appears to be stochastic (Figure 5) and each cluster can contain two (Figures 5A,B), three (Figure 5C), four (Figure 5D) or even more seam cell nuclei (Figure 5E). More often, certain seam cells are missing (Figure 4C), resulting in fewer than 16 SCM::GFP nuclei observed in wild-type animals (Figure 4B). Collectively, in the absence of MDF-2, the number of SCM::GFP nuclei is significantly decreased in young adult worms from 16 (observed in wild-type animals) to 14 in *Δmdf-2 *homozygotes (unpaired t-test, p = 2.96E-12) (Table 2). Furthermore, using *ajm-1::GFP *apical junction marker [33], we observed disruptions of seam syncytia in *Δmdf-2 *homozygote adult worms (data not shown), which further supports the importance of MDF-2 for proper seam cell development.

**Table 2:** The average number of SCM::GFP nuclei is altered in *mdf-2(tm2190*) mutants

Genotype	Hypodermal seam-cell nuclei (n)				
	L1	L2	L3	L4	Adult
Wild type	10.02 ± 0.08 (48)	15.76 ± 0.12 (25)	15.96 ± 0.04 (25)	16.04 ± 0.09 (25)	16.00 ± 0.08 (49)
*mdf-2(tm2190)*	9.75 ± 0.09* (59)	14.36 ± 0.30* (25)	14.08 ± 0.25* (25)	14.20 ± 0.39* (25)	14.28 ± 0.17* (64)

All of the strains are homozygous for the seam cell marker *wIs51 *(SCM::GFP). The number of SCM::GFP positive nuclei was scored 0 h after hatching (L1), two days after hatching (L4) and 12 h after L4 stage (young adults). Asterisk denotes significant difference when compared to wild type using unpaired students t-test analysis (p = 0.012 for L1 stage; p = 7.21E-5 for L2 stage; p = 2.57E-10 for L3 stage; p = 3.35E-5 for L4 stage and p = 2.96E-12 for adult stage.

During normal development, 10 precursor seam cells, H0-2, V1-6 and T, are formed during embryogenesis and are present at L1 after hatching. During L2, six of the 10 precursors undergo symmetrical division to produce additional seam cells totaling 16 seam cells at the end of L2 and beyond [34]. Therefore, the seam cell defects observed in *mdf-2(tm2190*) young adult worms could be either due to defective embryonic cell divisions, or alternatively, defective postembryonic divisions. In order to address these two possibilities, we scored the number of seam cell nuclei in newly hatched wild-type and *Δmdf-2 *L1 larvae. The wild-type animals analyzed had an average number of 10.02 SCM::GFP nuclei per side (range 9-11) (Table 2). Similarly, the majority of the *Δmdf-2 *newly hatched larvae had 10 SCM::GFP positive nuclei with 9.75 average and 8-11 range (Table 2). Although, unpaired students t-test analysis revealed a significant difference (p = 0.012), both the quantitative and qualitative defects observed in *Δmdf-2 *newly hatched larvae were much less severe than defects observed in L4 (unpaired t-test, p = 3.35E-5) or adults (unpaired t-test, p = 2.96E-12) (Table 2). Therefore, we conclude that MDF-2 plays an important role in postembryonic seam cell development.

Recently, it was reported that MDF-1 plays an important role in nutrient-deprivation induced somatic cell arrest [35]. Namely, it was found that hemizygosity of *mdf-1 *causes an increase in seam cell numbers from 10, observed in wild-type L1 worms starved for four days, to between 12 and 17 in more than half of the *mdf-1(gk2)/*+ L1 worms. To analyze the ability of *mdf-2(tm2190*) hemizygotes to arrest the proliferation during L1 diapause, we starved wild-type and *Δmdf-2/+ *hatchlings for four days. Subsequent analysis of the seam cells revealed that neither wild-type (n = 25) nor *Δmdf-2/+ *(n = 25) larvae had more than 11 SCM::GFP-positive nuclei, indicating starvation-induced L1 larval arrest. Thus, unlike MDF-1, MDF-2 component of the SAC does not seem to be required for starvation-induced somatic cell cycle arrest.

### The seam cell defect of *mdf-2(tm2190*) is due to defects in the proliferative seam cell division

The seam cells have stem cell-like properties and divide four times in developing larva for self-renewal maintenance, expansion, and to produce differentiated cells [30]. Six out of 10 embryonic seam cells, H1, V1-V4 and V6, undergo self-renewal expansion division at L2, resulting in an increase in the number of seam cells to 16 [30] (Figure 4B). To determine if the seam cell defect observed in *Δmdf-2 *homozygotes is due to a defect in proliferative cell division, we determined the number of SCM::GFP positive nuclei at late L2 and L3. We observed a mean of 14.36 (n = 25) seam cell nuclei at late L2 in the *Δmdf-2 *homozygotes (wild-type 15.76) and a mean of 14.08 (n = 25) seam cell nuclei at L3 in the *Δmdf-2 *homozygotes (wild-type 15.96), which is not significantly different from the number of SCM::GFP nuclei observed in later stages of the *Δmdf-2 *homozygotes (unpaired t-test, p = 0.7 and p = 0.8, respectively) (Table 2). These data demonstrate that the seam cell defect observed in *Δmdf-2 *homozygotes is most likely due to cell division defects at L2.

We next examined whether reduction of seam cell number could be attributed to failure of cell cycle progression of specific seam cells (H0-2, V1-V6 or T). We counted how often the observed seam cell defect is a consequence of failure of cell cycle progression of one particular cell (Figure 4C). Our analysis only includes unambiguous instances, where the identity of the defective nuclei could be determined (Figures 5 and 6). The cases where the identity of the defective seam nucleus is ambiguous, as in Figure 6B, were excluded from the analysis. We observed defects in all of the seam cells, H0-2, V1-V6 and T (Figure 4C), suggesting that failure of cell division affects all the cells in the seam cell linage. However, the frequencies of defects are different between the seam cells. For example, H0 seam cell defect was observed only once in 251 animals scored (Figure 4C). The H0 cells are the only cells, from the seam cell lineage, that do not undergo postembryonic division, further confirming the previous findings that the seam cell defect observed in *Δmdf-2 *homozygotes is mainly due to postembryonic defects. Similarly, H2, V5 and T cell defects were rarely observed (Figure 4C). In contrast, frequent defects were observed in the six seam cells, H1, V1-V4 and V6 that undergo expansion division to generate an additional six seam cells at L2 and beyond. These data support the findings that seam cell defects likely arise in L2 *Δmdf-2 *homozygotes. Furthermore, we quantified extra seam cell nuclei (Figures 5 and 4C) versus missing seam cell nuclei (Figures 6 and 4C) and, as expected, we observed that reduction of the number of SCM::GFP positive nuclei is a much more common event (Figure 4C). Representative images of seam cell reduction due to a failure of cell cycle progression of a particular lineage are shown in Figure 6. Together, these data indicate that seam cell defects in the absence of MDF-2 are mainly attributed to cell proliferation failure at L2 which randomly affects H1, V1-V4 or V6 seam cells.

### The seam cell reduction in *mdf-2(tm2190*) is not likely to be caused by *ced-3 *dependent cell death

It is possible that the reduction of number of seam cells in *Δmdf-2 *worms is caused by cell damage followed by apoptotic cell death. CED-3 is a member of the caspase family of cystein proteases that is required for cell death in *C. elegans *[36]. To determine whether apoptotic cell death could account for loss of seam cells, we constructed *ced-3(n717) unc-26(e205) mdf-2(tm2190*) in which there is no cell death. We found that *ced-3(n717) unc-26(e205*) mutants do not affect seam cell development, as on average 15.92 seam cell nuclei were observed in young adults (unpaired t-test, p = 0.3, when compared to wild type young adults) (Table 3). Furthermore, we found that *ced-3(n717) unc-26(e205) mdf-2(tm2190*) animals had similar numbers of seam cell nuclei (on average 14.27; unpaired t-test, p = 0.98) as *mdf-2(tm2190)*, suggesting that *ced-3 *dependent cell death is unlikely to be responsible for seam cell loss in the *tm2190 *background.

**Table 3:** The average number of SCM::GFP positive nuclei observed in different genetic backgrounds

Genotype	Hypodermal seam-cell nuclei	Range	n (sides)
Wild type	16.00 ± 0.08	15-17	49
*mdf-2(tm2190)*	14.28 ± 0.17	8-19	64
*ced-3(n717) unc-26(e205)*	15.92 ± 0.08	15-17	48
*ced-3(n717) unc-26(e205) mdf-2(tm2190)*	14.27 ± 0.25	8-17	55
*fzr-1(ok380)*	15.98 ± 0.07	15-17	50
*fzr-1(ok380)*; *mdf-2(tm2190)*	14.82 ± 0.26	10-20	50
*fzy-1(h1983)*	16.04 ± 0.12	14-18	50
*fzy-1(h1983)*; *mdf-2(tm2190)*	16.08 ± 0.11	14-18	50

All of the strains are homozygous for the seam cell marker *wIs51 *(SCM::GFP). The number of SCM::GFP positive nuclei was scored in young adult hermaphrodites. Note that data for wild-type and *mdf-2(tm2190*) young adults are duplicates of the data shown in Table 2.

### Absence of FZR-1 enhances sterility of *mdf-2 *mutants without causing any effect on seam cell development

**Figure 7:**
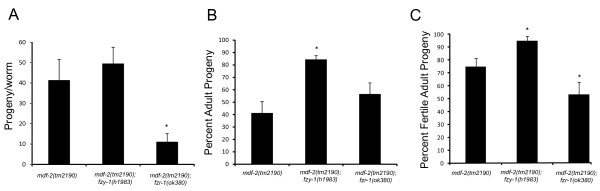
**Genetic interactions between *mdf-2(tm2190)/MAD2*, *fzr-1(ok380)/CDH1 *and *fzy-1(h1983)/CDC20***. (A) Brood size - scored as total number of eggs laid by a worm. (B) Viability - scored as percent of the progeny that developed to adulthood. (C) Fertility - percent of adult progeny that are fertile.

During postembryonic development, seam cell division is regulated at the G1 to S phase progression by a cascade of regulatory factors that include LIN-35/Rb, FZR-1/Cdh1, and CKI-1 [37-42]. As LIN-35 and FZR-1 act redundantly to control the G1 to S phase progression, seam cell proliferation appears to be normal in *lin-35 *and *fzr-1 *single mutants, while extensive hyperproliferation is observed in *lin-35*; *fzr-1 *double mutants [39]. Furthermore, *lin-35 *and *fzr-1 *single mutants rescue postembryonic seam cell defects in *bro-1 *single mutants [42]. *bro-1 *is the *C. elegans *CBFβ homolog that is required for the normal proliferation and differentiation of seam cells [42]. To determine whether or not *lin-35 *and *fzr-1 *mutants play a role in the defective postembryonic cell proliferation in the *mdf-2(tm2190*) background, we examined genetic interactions by constructing *lin-35(rr33)*; *mdf-2(tm2190*) and *fzr-1(ok380)*; *mdf-2(tm2190*) double mutants. We found that 100% of the *lin-35(rr33)*; *mdf-2(tm2190*) double mutants are sterile, making the analysis of seam cell development difficult. We also found synthetic enhanced interaction between *fzr-1(ok380*) and *mdf-2(tm2190*) mutants (Figure 7). The *ok380 *deletion removes 442 nucleotides between intron 3 and exon 3 and is predicted to result in truncated FZR-1, which may or may not be functional. *fzr-1(ok380*) homozygotes can be easily propagated and exhibit no major developmental abnormalities. As reported previously, *mdf-2(tm2190*) homozygotes can be maintained at 20°C indefinitely but display a severely reduced brood size of approximately 40 progeny/worm (Figure 7A) of which only 40% develop into adults [12] (Figure 7B). Once we constructed *Δfzr-1*; *Δmdf-2 *homozygotes, we immediately observed that these worms are extremely difficult to propagate due to the small number of progeny that reach adulthood. Our detailed analysis of *Δfzr-1*; *Δmdf-2 *double mutants revealed that they have significantly reduced brood sizes (~11 progeny per worm) (Figure 7A) and significantly reduced numbers of fertile adults (~50% of all adult progeny are fertile), resulting in only two or three fertile adult progeny per hermaphrodite compared to about 10 to 15 fertile adults produced by *Δmdf-2 *homozygotes (Figure 7). Furthermore, we observed that while *Δmdf-2 *homozygotes displayed CIN as determined by *h*igh *i*ncidence of *m*ales (Him) phenotype (~3% of the adult progeny are males; n = 252), *Δfzr-1 *increases this chromosome instability to ~6% (n = 107 adult progeny observed).

Even though *Δfzr-1*; *Δmdf-2 *double mutants are difficult to grow, we collected enough adult progeny for analysis of postembryonic seam cell proliferation. As expected, we found that *Δfzr-1 *homozygotes (n = 50) had on average 15.98 SCM::GFP nuclei (Table 3) not significantly different from wild-type (unpaired t-test, p = 0.8). However, we found that *Δfzr-1 *had no effect on seam cell proliferation in the *mdf-2(tm2190*) background as *Δfzr-1*; *Δmdf-2 *double mutants had on average 14.82 (Table 3) seam cell nuclei not significantly different from the *Δmdf-2 *animals (unpaired t-test, p = 0.6). Taken together, these data suggest that although *mdf-2 *displays synthetic lethality and enhanced phenotype with *lin-35 *and *fzr-1*, this pathway is unlikely explanation for postembryonic cell proliferation defect observed in the absence of MDF-2 spindle-checkpoint using the seam cell lineage.

### Hypomorphic mutant *fzy-1(h1983*) partially suppresses lethality of *mdf-2 *mutants and completely rescues seam cell defects

The hypomorphic mutant allele of *fzy-1*,*h1983*, was isolated from the screen for suppressors of the *mdf-1(gk2*) lethal phenotype in search for additional components that function in the metaphase-to-anaphase transition [43]. The *h1983 *allele is a missense mutation and the resulting FZY-1D433N mutant protein cannot properly bind the APC/C substrate IFY-1 (securin) [43]. Subsequently, it has been shown that *fzy-1(h1983*) rescues *mdf-1(gk2*) lethality likely by delaying anaphase onset because the duration of mitosis in *fzy-1(h1983*) early-stage embryos is extended, presumably due to an increased level of securin [44]. While the main function of MDF-1 may be regulation of APC/C activity [43,44], the precise role for MDF-2 is currently unknown.

*fzy-1(h1983*) homozygotes can be easily propagated and the strain exhibits a slight decrease in the brood size and an increase in incidence of males with no apparent abnormalities in growth or morphology [43]. To determine whether *fzy-1(h1983*) can rescue lethality of the *mdf-2(tm2190)*, we constructed *fzy-1(h1983)*; *mdf-2(tm2190)*. We observed that *fzy-1 *has no significant effect on brood sizes of *Δmdf-2 *homozygotes (Figure 7A). However, *fzy-1*; *Δmdf-2 *worms produce on average 85% progeny that develop into adults, compared to ~40% observed for *Δmdf-2 *homozygotes (Figure 7B). Furthermore, the majority (~95%) of *fzy-1*; *Δmdf-2 *adult progeny are fertile (Figure 7C), suggesting that *fzy-1(h1983*) can suppress the sterility caused by the absence of MDF-2. Also, we observed that *fzy-1 *decreases incidence of males from ~3% observed in the *Δmdf-2 *homozygotes to ~0.8% observed in double mutants. Together, these data further confirm that like MDF-1, MDF-2 regulates APC/C^CDC20 ^activity during development.

Next, we examined if *fzy-1(h1983*) has an effect on seam cell development. Interestingly, we found that *fzy-1(h1983*) homozygotes had on average 16.04 (Table 3) seam nuclei not significantly different from wild-type animals (unpaired t-test, p = 0.8). Furthermore, seam cell development in *fzy-1; Δmdf-2 *double mutants appeared to be completely normal (Table 3). Namely, *fzy-1; Δmdf-2 *double mutants had on average 16.08 (Table 3) seam cell nuclei not significantly different from the wild-type or *fzy-1(h1983*) homozygous animals (unpaired t-test, p = 0.8). In addition, the majority of the analyzed *fzy-1; Δmdf-2 *young adults had 16 evenly spaced and aligned SCM::GFP nuclei. These results suggest that MDF-2 plays an important role in postembryonic seam cell proliferation by inhibiting the activity of the APC/C^CDC20^.

## Discussion

In this work we have examined for the first time *in vivo *spatiotemporal expression profiles of eight spindle-checkpoint genes in *C. elegans*. Among these eight genes, five are conserved from yeast to human (*mdf-1*, *mdf-2*, *san-1*, *bub-1 *and *bub-3*) [9-13], while three are conserved in higher eukaryotes (*hcp-1*, *hcp-2 *and *rod-1*), including *C. elegans *[12,16,45]. Our study focused on analysis of the expression patterns by using extra-chromosomal arrays. To maximally reduce the effect of mosaicism, the known caveat of this approach, we analyzed a large number of animals for each developmental stage, and recorded the tissues and cells where GFP expression was consistently observed. On the other hand, we found the mosaicism to be beneficial for a better identification of tissues where GFP is expressed. When promoters drive GFP expression in more than one tissue types, then expression restricted to only small groups of cells, due to loss of the array, offers more confident identification of these tissues. Also, GFP expression is a sensitive technique which is important for SAC gene expression analysis because generally SAC genes do not produce an abundant number of transcripts. Concatamer arrays were previously suggested to be a sensitive tool for detecting gene expression for genes with low levels of transcription [23]. We confirmed the sensitivity of this approach when we generated a p*mdf-2::GFP *stable line using MosSCI [31]. This stable line had very low GFP signal intensity and required long exposure times for the expression to be observed.

The 5' DNA sequences selected as containing putative promoters of the SAC genes displayed common early embryonic activities in the majority, if not all, of the rapidly dividing embryonic cells. This finding is consistent with the known roles of the checkpoint genes in cell division. We expected this result because of the fact that 556 of the 959 somatic cells present in adult hermaphrodite are generated during embryogenesis [30]. Furthermore, our observations of early embryonic expression is supported by published analyses which used antibodies against some of the SAC gene products [9,11,15,27,45]. Thus, it is likely that these transcriptional fusions recapitulate endogenous SAC gene promoter activities. Importantly, this common "ubiquitous" expression of SAC genes (including *mdf-1*) during early embryogenesis, suggests that expression of *mdf-1*, the only one located within an operon, has to be driven by the internal promoter (Additional file 1, Figure S1). Thus, the *mdf-1 *containing operon is likely a "hybrid operon" [28].

*czw-1 *(known as ZW10 in other organisms) was also included in our study; however, analysis of two different constructs did not reveal any detectable GFP expression. It is possible that expression of the analyzed transgenes was either too low for visible detection, germline specific, conditional, or that regulatory elements of this gene are located in regions not included by our putative promoter selection criteria.

In contrast to expression in embryos, postembryonic expression of SAC genes in *C. elegans *is more localized. During the four larval stages in a hermaphrodite, the 53 undifferentiated somatic blast cells generate an additional 403 somatic nuclei [30]. The somatic blast cell divisions generate somatic gonad, muscle, coelomocytes, nerves, hypodermis and intestine [30,46,47]. If all of the checkpoint genes played the same role in postembryonic development, one would expect to observe the same expression patterns for the SAC genes. However, our analysis revealed that checkpoint promoters generally dictate differential postembryonic expression patterns. For example, it is very interesting that *mdf-1^internal ^*and the *rod-1 *promoters drive GFP expression exclusively in intestine after embryogenesis, while the *hcp-1 *promoter drives GFP expression in multiple tissues (Table 1). These findings suggest distinct, yet overlapping, roles of the checkpoint genes in postembryonic development and provide an excellent resource for further research. Recently, staining of newly hatched L1 larva with anti-MDF-1 antibody revealed specific localization of MDF-1 to intestinal cells and germ cell precursors [35], which further supports our findings from using the transcriptional reporter system. We did not observe expression in germ cell precursors or any other germ cells possibly due to silencing of concatamer transgenes in the germinal gonad.

An unexpected finding from our analysis was tissue-specific expression of SAC genes in late L4 and adults that contain no somatic cells destined to divide. Considering that tissue-specificity observed in these stages was similar to the tissue-specificity observed in larval stages, it is possible that the observed patterns reflect longer turnover times for the GFP carried over from earlier larval stages [23]. On the other hand, it is possible that 5' upstream sequences used in our analysis do not include important "repressor" elements that are required for proper expression of SAC genes. Alternatively, it may be that SAC genes have roles in these adult tissues that remain to be uncovered.

We have found that spindle-checkpoint genes reveal an intriguing co-expression in hypodermal seam cells. This finding prompted us to use the seam cell lineage to test the functional importance of the checkpoint for proper postembryonic cell proliferation. Here, we demonstrated that the knockout allele, *tm2190*, of *mdf-2 *results in defective seam cell development that is mainly attributed to seam cell proliferation failure at L2. In the absence of MDF-2, on average 14 seam cell nuclei were observed instead of expected 16. The number of SCM::GFP nuclei per side of an animal ranges from 8 to 19 in the absence of MDF-2 (Table 3). While the majority of the Δ*mdf-2 *homozygotes contains less than expected 16 seam cell nuclei per side in young adults, we also observed animals that had more than 16 seam cell nuclei (Figure 5), which could be attributed to defective cell division. The results presented in this paper provide the first evidence that embryonic cell divisions are more tolerant to the loss of SAC, in particular MDF-2, than postembryonic cell divisions, as determined using the seam cell lineage. Furthermore, we show that the importance of MDF-2 for proper seam cell proliferation depends on its regulation of APC/C^CDC20^. The seam cell defect in Δ*mdf-2 *homozygotes cannot be explained by cell damage followed by caspase-dependent apoptotic cell death, since *ced-3 *mutant had no effect on seam cell defect in Δ*mdf-2 *worms. Furthermore, *fzy-1(h1983*) rescued all of the Δ*mdf-2 *phenotypes, except for the brood size. On the other hand, G1 phase regulators, LIN-35 and FZR-1, when defective affect only brood size in the absence of MDF-2. The analysis presented here, using the Δ*mdf-2*, serves as an excellent model for further studies on effects of a defective SAC on development of different tissues in a multicellular organism.

A striking emerging pattern is that essentially all SAC genes are expressed in intestine and hypodermis. SAC components MDF-2 [9] and MDF-1 [35] have previously been observed to be localized to gut cells by using antibody staining. Endoreduplication, also known as endoreplication, is a process in which S phases are not followed by mitosis. This process gives rise to cells with extra copies of chromosomes, permitting amplification of the genome in specialized cells. In humans, these include hepatocytes, cardiomyocytes and megakaryocytes [48]. In *C. elegans*, two tissues are polyploid: the hypodermis and the intestine [49]. Our finding of co-expression of SAC genes in these tissues may suggest a possible role of these genes in the process of endoreduplication in *C. elegans*. Furthermore, our findings clearly suggest that SAC genes are differentially regulated at the transcription level at different developmental stages.

## Conclusion

We have examined for the first time *in vivo *spatiotemporal expression profiles of eight conserved spindle assembly checkpoint genes in *C. elegans*. Our comprehensive analysis revealed that all of the SAC gene promoters displayed common early embryonic activities in the majority, if not all, of the rapidly dividing embryonic cells. Furthermore, we found that all of the SAC gene promoters drive tissue specific postembryonic expression. The expression patterns differ between the SAC genes; the majority of the SAC genes co-express in hypodermal seam cells and gut cells. These findings suggest that the SAC components may have distinct roles in postembryonic development which could be different from their role in mitosis. Furthermore, our analysis provides an important starting point for analysis of the checkpoint roles in development of a multicellular eukaryote that may offer explanation for distinct phenotypic consequence upon inactivation of different SAC genes. It is extremely important to determine how defects in different SAC components affect cell proliferation, cell fate determination and cell differentiation in a multicellular organism.

## Methods

### *C. elegans *strains, alleles and culturing

The Bristol strain N2 was used as the standard wild-type strain [50]. The following mutant alleles were used in this work: *dpy-5(e907), mdf-1(gk2), mdf-2(tm2190)*, *ced-3(n717), unc-26(e205), lin-35(rr33)*; *fzr-1(ok380*) and *fzy-1(h1983)*. The wls51 (SCM::GFP) strain JR667 was used to visualize the seam cell nuclei in wild-type worms and the mutant backgrounds. The strains were obtained from the Caenorhabditis Genetics Center (University of Minnesota, Minneapolis, MN) unless otherwise stated. The following transgenic strains were generated: JNC104 [*dpy-5(e907*) I; dotEx104 (Y69A2AR.30::GFP + pCeh361)]; JNC105 [*dpy-5(e907*) I; dotEx105 (C50F4.13::GFP + pCeh361)]; JNC106 [*dpy-5(e907*) I; dotEx106 (ZC328.4::GFP + pCeh361)]; JNC107 [*dpy-5(e907*) I; dotEx107 (Y54G9A.6::GFP + pCeh361)]; JNC108 [*dpy-5(e907*) I; dotEx108 (R06C7.8::GFP + pCeh361)]; JNC109 [*dpy-5(e907*) I; dotEx109 (ZK1055.1::GFP + pCeh361)]; JNC110 [*dpy-5(e907*) I; dotEx110 (T06E4.1::GFP + pCeh361)]; JNC111 [*dpy-5(e907*) I; dotEx111 (C50F4.11::GFP + pCeh361)]; JNC112 [*dpy-5(e907*) I; dotEx112 (F20D12.4^2101 bp^::GFP + pCeh361)]; JNC113 [*dpy-5(e907*) I; dotEx113 (F20D12.4^3000 bp^::GFP + pCeh361)]; JNC114 [*dpy-5(e907*) I; dotEx114 (F55G1.4::GFP + pCeh361)]; JNC115 [*dpy-5(e907*) I; dotEx115 (C50F4.11^1332 bp^::GFP + pCeh361)]; JNC116 [*dpy-5(e907*) I; dotIs104 (Y69A2AR.30::GFP + pCeh361)]; JNC117 [*unc-119(ed3*) I; dotSi104 II (Y69A2AR.30::GFP + *unc-119(+)*]. Animals were maintained using standard procedures [50].

### Generation of pSAC::GFP transgenic animals

The promoter::GFP constructs were generated using the "PCR stitching" technique [19]. The PCR experiments were designed to amplify and fuse 5' sequence immediately upstream of the predicted ATG initiator site for a targeted gene to an adjacent upstream gene. All of the primers were designed semi-manually with the aid of primer3 [51] and used in standard PCR procedures to amplify putative SAC gene promoters from *C. elegans *N2 (Bristol) single worm lysates. These amplicons were then fused to the PCR products containing *gfp *sequence and *unc-54 *3'UTR from pPD95.75 (developed by Dr. Andrew Fire http://www.addgene.org/pgvec1?f=c&identifier=1494&atqx=%C2%A0pPD95_75&cmd=findpl). For fusion PCR reactions we used Phusion (NEB) high-fidelity DNA polymerase. All of the promoter::GFP fusion PCR products were confirmed by sequencing before injection. We injected fusion PCR products, without purification, into the gonad of young adult hermaphrodites of CB00907 at a concentration of 10 ng/μL together with 100 ng/μL *dpy-5*(+) plasmid (pCeh361) in 1XTE buffer to generate extrachromosomal arrays. On average, 25-30 P_0 _*dpy-5(e907*) hermaphrodites were injected with each pSAC::GFP construct. Rescued Dpy-5 mutant phenotype was indicative of transformants. These wild-type looking F_1 _progeny were plated individually and screened for the presence of wild-type F_2 _progeny. On average, we obtained three to five lines yielding at least 30% wild-type progeny. Aware of the mosaicism issues associated with extrachromosomal concatamer arrays, we analyzed at least 30 replicates for each developmental stage. Once these lines were genotyped and confirmed to have similar expression patterns, one line for each construct was frozen and kept as a transformed stock. Genotyping was performed using promoter specific primer and GFP specific primer.

### *In vivo *analysis and imaging of pSAC::GFP transgenic lines

For each transgenic line, we prepared mixed-staged population of worms and immobilized them in 100 mM sodium azide (in water) immediately before imaging. Initially, worms were analyzed using a Zeiss Axioskop equipped with QImaging camera to confirm the consistency of expression patterns between the transgenic lines. Then more detailed analysis, which involved taking stacks of confocal images with 0.2-0.5 μm between focal planes, was performed using Quorum WaveFX Spinning Disk system mounted on a Zeiss Axioplan microscope. All images were taken at 400X, image acquisition and analysis was performed using a Volocity software package (Improvision, Coventry, England).

### Viability measurement

For all the double and single mutants, five L4 wild-type looking worms were individually plated at 20°C. The worms were transferred to fresh plates every 12 hours and the plates were scored. Total numbers of eggs laid defined the brood sizes. The eggs that did not hatch in 24 hours were scored as embryonic arrest. The eggs that hatched but did not reach adulthood were scored as larval arrest. The progeny that developed to adulthood were scored for incidence of males. The percent fertility was determined by individually plating all progeny that developed to adulthood. All of the single and double mutants were then analyzed in a SCM::GFP background for number of seam cells by using Zeiss Axioskop equipped with QImaging.

## Abbreviations

APC/C: *a*naphase *p*romoting *c*omplex or *c*yclosome; CIN: *c*hromosome *in*stability; DIC: *d*ifferential *i*nterference *c*ontrast; GFP: *g*reen *f*luorescent *p*rotein; Him: *h*igh *i*ncidence of *m*ales; MosSCI: *Mos*1-mediated *s*ingle-*c*opy *i*nsertion; RNAi: *RNA i*nterference; SAC: *s*pindle *a*ssembly *c*heckpoint.

## Authors' contributions

NC and MTG conceived of the study. MTG designed the experiments, performed the expression analysis, seam cell analysis, genetic interaction analysis and wrote the manuscript. JW participated in seam cell analysis. JSSC participated in spatiotemporal expression analysis. DT generated transgenic strains. NC and DLB helped to draft the manuscript. All authors read and approved the final manuscript.

## Supplementary Material

Additional file 1 **Figure S1: Putative *mdf-1/MAD1 *promoter activities.** (A) 295 bp of the 5' regulatory region immediately upstream of *his-35*, the first gene in the *mdf-1 *containing operon, drives localized GFP expression from the embryonic stage. (*Left*) GFP images; (*Right*) DIC images. (B) The internal promoter is the 543 bp sequence between *his-35 *and *mdf-1*. This promoter drives ubiquitous GFP expression in the embryo as expected. (C) 1332 bp of the 5' regulatory region upstream from the ATG initiator site in *mdf-1 *- extending to the operon adjacent upstream gene (*his-41*) - results in ubiquitous GFP expression in embryos, similar to the internal *mdf-1 *promoter expression pattern. Click here for file
