# Supplementary figures and images for: Spindle assembly checkpoint genes reveal distinct as well as overlapping expression that implicates MDF-2/Mad2 in postembryonic seam cell proliferation in Caenorhabditis elegans
Source: BMC Cell Biol. 2010 Sep 21;11:71. doi: 10.1186/1471-2121-11-71 (PMC2955571; doi:10.1186/1471-2121-11-71)

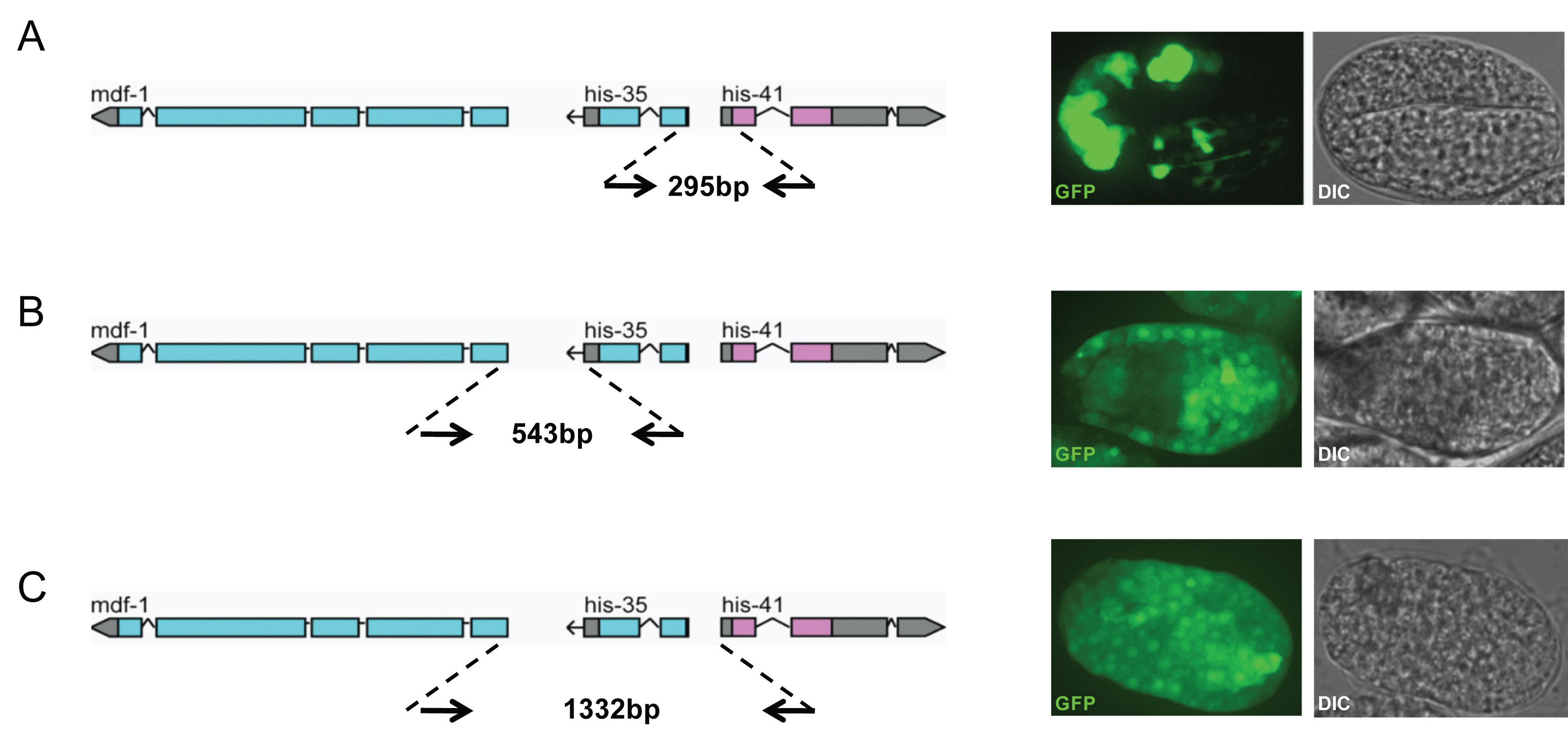

Supplement: Additional file 1 — Figure S1: Putative mdf-1/MAD1 promoter activities. (A) 295 bp of the 5' regulatory region immediately upstream of his-35, the first gene in the mdf-1 containing operon, drives localized GFP expression from the embryonic stage. (Left) GFP images; (Right) DIC images. (B) The internal promoter is the 543 bp sequence between his-35 and mdf-1. This promoter drives ubiquitous GFP expression in the embryo as expected. (C) 1332 bp of the 5' regulatory region upstream from the ATG initiator site in mdf-1 - extending to the operon adjacent upstream gene (his-41) - results in ubiquitous GFP expression in embryos, similar to the internal mdf-1 promoter expression pattern. [file 1471-2121-11-71-S1.TIFF]
